# The Role of Next-Generation Sequencing in Precision Medicine: A Review of Outcomes in Oncology

**DOI:** 10.3390/jpm8030030

**Published:** 2018-09-17

**Authors:** Margaret Morash, Hannah Mitchell, Himisha Beltran, Olivier Elemento, Jyotishman Pathak

**Affiliations:** 1 Division of Health Informatics, Department of Healthcare Policy and Research, Weill Cornell Medicine, New York, NY 10065, USA; ham2026@med.cornell.edu (H.M.); jyp2001@med.cornell.edu (J.P.); 2 Division of Medical Oncology, Weill Cornell Medicine and New York-Presbyterian Hospital, New York, NY 10065, USA; hip9004@med.cornell.edu; 3 Meyer Cancer Center, Weill Cornell Medicine, New York, NY 10065, USA; ole2001@med.cornell.edu; 4 Englander Institute for Precision Medicine, Weill Cornell Medicine-New York Presbyterian Hospital, New York, NY 10065, USA; 5 Institute for Computational Biomedicine, Weill Cornell Medicine, New York, NY 10065, USA

**Keywords:** precision medicine, next generation sequencing, oncology, patient outcomes, health insurance coverage

## Abstract

Precision medicine seeks to use genomic data to help provide the right treatment to the right patient at the right time. Next-generation sequencing technology allows for the rapid and accurate sequencing of many genes at once. This technology is becoming more common in oncology, though the clinical benefit of incorporating it into precision medicine strategies remains under significant debate. In this manuscript, we discuss the early findings of the impact of next-generation sequencing on cancer patient outcomes. We investigate why not all patients with genomic variants linked to a specific therapy receive that therapy and describe current barriers. Finally, we explore the current state of health insurance coverage for individual genome sequencing and targeted therapies for cancer. Based on our analysis, we recommend increased transparency around the determination of “actionable mutations” and a heightened focus on investigating the variations in health insurance coverage across patients receiving sequencing-matched therapies.

## 1. Introduction

**Figure 1:**
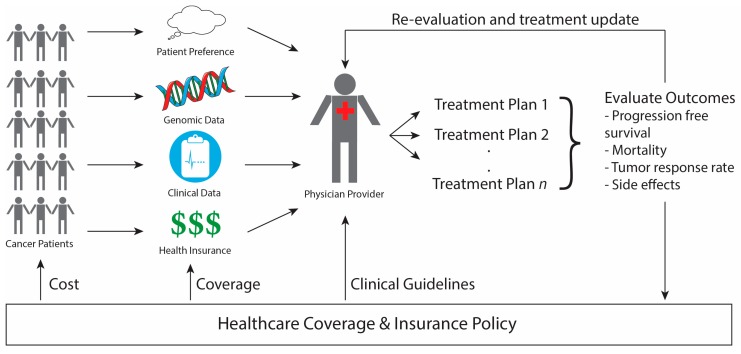
Outline of Precision Medicine in Oncology. Cancer patients have genomic, clinical, and insurance information that is evaluated by the physician, along with patient preferences, to design a potential treatment plan via shared-decision making. The patient’s outcomes are evaluated both to update their individual treatment plan as well as to inform future healthcare policy making. Once enough evidence amasses to show the clear benefit of a certain treatment, changes in healthcare policy affect (1) the clinical guidelines physicians consult in designing care, (2) the types of treatments that health insurance policies cover, and (3) the cost of treatment to the patient.

The speed, accuracy, and increasing affordability of next-generation sequencing (NGS) has helped spur the advent of precision medicine, which involves designing treatment based on a person’s disease-driving molecular alterations [1,2,3]. While NGS has been tested across multiple health care settings, its use is most advanced in oncology with physicians sequencing their patients’ tumors to match them to therapies designed to target the genetic alterations driving the tumor’s growth. We will refer to such therapies as sequencing-matched therapies. The overview of precision medicine in oncology presented in Figure 1 demonstrates how genomic data, clinical information, and patient preferences inform clinical decision making to improve outcomes by matching each patient with the therapy best suited to treat their cancer. The extent to which incorporating NGS into care improves patient outcomes, such as treatment response and disease-free survival, however, remains controversial. Furthermore, insurance coverage of NGS technology and sequencing-matched therapies is also under debate. Below we address these questions of clinical utility and health policy in NGS-guided care in oncology.

## 2. Precision Medicine in Oncology

### 2.1. The Promise

Several studies have shown the utility of NGS in identifying clinically actionable mutations in cancer patients. For example, the Genomics Evidence Neoplasia Information Exchange (GENIE), an international data-sharing consortium, estimated an actionability rate of 30% across several cancers [4]. That is, 30% of tumors sequenced in the GENIE consortium had a mutation that could be targeted by an existing targeted therapy.

Using sequencing results to match patients to a therapy based on their cancer’s genome has shown benefits in patient outcomes (summarized in Table 1). In Tsimberidou et al.’s Phase I study, advanced cancer patients given a treatment matched to their tumor mutations showed improved overall response rate (27% vs. 5%), time to treatment failure (median of 5.2 vs. 2.2 months), and survival (median of 13.4 vs. 9.0 months) when compared to patients who did not receive sequencing-matched therapy [5]. These metrics evaluate the change in tumor size; the time from the start of treatment to when a patient came off the study due to toxicity, disease progression, or death; and the time from the start of treatment until death or last follow-up, respectively. Similarly, Radovich et al. [6] reported that the progression free survival of patients with treatments matched to their DNA mutations, copy number variations, or mRNA levels was higher than that of patients receiving non-matched therapy (86 vs. 49 days). Progression free survival, another common evaluation statistic in oncology, measures the time between the start of treatment and the growth of the cancer. Additional studies have also reported improvements in progression free survival [7], overall survival [8,9], and tumor response [7,10] for patients on sequencing-matched therapy vs. non–matched.

There have also been advancements in developing drugs that target tumor-driving mutations identified by NGS. Le et al. [12] reported that PD-1 blockade treatment was effective across 12 different tumor types with “loss-of-function” mutations in the mismatch repair pathway. This trial led to the first USA food and drug administration (FDA)-approval for a drug (pembroluzimab) in 2017 to be given based solely on mutations and not tumor type—a purely precision medicine approach [13]. A similar histology-agnostic approach has also shown promise in a first-in-human study by Drilon et al. [14], which reports the potential of using LOXO-195 across tumor types, dependent on specific gene fusions. As more studies are done, physician education through tumor board discussions and reviews accessible to non-experts are needed to increase understanding and comfort utilizing genomic sequencing and sequencing-matched therapies.

### 2.2. The Limitations

While the above reports show the utility of incorporating NGS into cancer care, there are no randomized controlled trials supporting a NGS-based treatment approach [15,16,17]. Since NGS can identify so many diagnostic sub-categories, however, it makes it exceedingly difficult to accrue sufficiently large populations to power a randomized controlled trail for each cancer sub-type NGS can identify. Indeed, understanding these limitations, the FDA approved the first precision medicine therapy, pembroluzimab, without evidence from a randomized controlled trial [13]. It is important to note, however, that the only precision medicine randomized controlled trial to date saw no benefit in patient outcomes when using NGS to match patients to targeted treatments regardless of cancer type [11]. More specifically, this phase II trial included 195 advanced cancer patients and saw no difference in progression free survival between the control group, who were treated according to their physician’s choice, and the test group, who were matched with therapies based on molecular profiling [11]. This partially reflects the complexity of treating advanced cancer patients whose tumors are genetically highly heterogeneous, meaning that different cells within the same tumor may have different mutations. Nonetheless, the study raised important questions about the clinical utility of using drugs outside of their recommended setting based on sequencing results alone.

Another limitation of current efforts to evaluate NGS precision medicine strategies is the variation across sequencing-matched and non-matched groups within a single study, and variations in populations in different studies. For example, specific cancer types, like metastatic melanoma, will have a higher rate of actionable mutations than, for example, prostate cancer due to the high prevalence of BRAF mutations in melanomas [18,19]. Furthermore, whether patients with targetable mutations have cancer that is inherently less aggressive or easier to treat remains to be explored. While the population varies across studies in Table 1, many studies nonetheless indicate that using sequencing results to inform patient treatment plans shows clinical benefit.

The other caveat to the success of using NGS in cancer care lies in the small percentage of sequenced patients with “actionable mutations” that are ultimately treated with a sequencing-matched therapy (shown in Table 2). This phenomenon is seen across several studies [8,10,20,21,22,23] and, while there are practical barriers that preclude patients from receiving sequencing-matched therapy, it raises questions about the clinical utility of the “actionable mutation” metric. As there is no standard definition of an “actionable mutation,” it may be that some studies apply a much broader interpretation, including mutations that impact the patient’s prognosis or indicate an inherited cancer syndrome [24]. In these cases, many patients who are said to have actionable mutations may not in actuality be able to use their sequencing information to match them to a cancer therapy [15]. Thus, it is important for studies to be transparent and precise about how they determine whether a mutation is actionable or not, and also to draw clear distinctions between different categories of mutations and their potential impact. For example, the levels of actionable mutations used in the GENIE study were clearly defined starting with level 1 gene alterations indicative of treatment with standard of care therapy in the same cancer type to level 3B indicative of promising investigational therapy in a different cancer type [4].

## 3. Barriers to Individualized Treatment

Aside from the discrepancy in the “actionable mutation” terminology, there are practical barriers that help explain the large drop off (shown in Table 2) between patients with actionable mutations and patients receiving sequencing-matched therapy. We discuss some of these challenges below.

### 3.1. Physician Interpretation and Patient Preference

Some physicians may not feel comfortable interpreting sequencing results or directing their patients’ therapy based on genomic data [25]. In a recent survey of 46 oncology providers at Mayo Clinic (Rochester, MN, USA), 52% of providers were slightly uncomfortable or not at all comfortable interpreting information from a genomic test [24]. Individual physician’s lack of familiarity with interpreting genomic results can be partially accounted for by tumor boards. These boards bring together experts in genomic sequencing and oncology to identify actionable therapeutic targets and provide a forum for discussion of complex cases between scientists and physicians. Another factor preventing patients from getting sequencing-matched therapy is that some patients succumb to cancer before receiving the sequencing results, or else have reached a stage in their disease progression where they elect to stop treatment and pursue hospice care. A study by Bryce et al. reported that 65%, or 22 out of 34 eligible patients, either passed away or pursued comfort measures instead of proceeding with sequencing-matched therapies [24]. In these cases, implementing sequencing earlier, or pre-emptively [26,27], in patients’ cancer care would likely allow them the opportunity to direct their treatment based on their sequencing results.

### 3.2. Eligibility for and Access to Care Options

Patients’ access to clinical trials is restricted by the number and location of trials. In addition to availability, patients—especially the advanced-stage cancer patients commonly included in cancer sequencing studies—are often not eligible for clinical trials due to previous treatment or comorbidity. This is true for the ongoing National Cancer Institute (NCI) Molecular Analysis for Therapy Choice (MATCH) study (clinicaltrials.gov identifier NCT02465060), which reported in their interim analysis in May of 2016 that, of 56 enrolled patients with a mutation that matched one of the ten available treatment arms, only 33 met the eligibility criteria [28]. By July of 2017, the study met their goal of sequencing tumor samples from 6000 patients, of which 5560 or 93% were successfully sequenced. While they do not have available data on the number of patients who met eligibility criteria, 992 (18%) were matched to a study arm, but only 689 (12%) ultimately enrolled in the study [29].

### 3.3. Cost and Insurance Coverage

#### 3.3.1. Patient Perspective

Other patients are unable to access care options because of the high cost of NGS and sequencing-matched therapies. Patients looking for tumor sequencing to help match them to a therapy outside of a research study, which often covers the cost of NGS, may struggle to get insurance to cover it. Many insurance companies cover companion diagnostic DNA tests, tests of specific genes that indicate whether treatment with a specific therapy is appropriate [30]. Reimbursement is much more limited and variable across providers for NGS technologies like whole-exome and genome sequencing, which provide information on a much broader range of genes, but which are often viewed as investigational and suitable for research instead of clinical care [31,32].

To get access to sequencing-matched therapies, patients often enroll in clinical trials. Outside of clinical trials, a patient can receive a targeted therapy as long as the drug has been approved by the FDA. Receiving these therapies outside of their original indication, such as at a different dose or frequency or in patients with a different cancer type or age range, is called off-label use. Such off–label drug use is very common in routine cancer treatment, with a recent review reporting that as many as 71% of adult cancer patients receive at least one off-label cancer therapy [33,34]. Indeed, Medicare provides coverage for off-label usage of FDA-approved drugs based on the recommendation of five approved compendia [35]. Not all insurers in the U.S. take the same approach as Medicare, however, and most private insurers decide which off-label drugs they will cover on a case-by-case basis, meaning that patient access to these drugs may be highly variable [36]. While the issue of variable insurance coverage is not pertinent to single-payer systems such as those found in Canada and England, the concerns over the cost of genomic sequencing and use of off-label therapies applies in all systems.

#### 3.3.2. Policy Implications

There has been a recent call for insurance to cover NGS-based tests so that researchers and physicians can amass enough information to identify all clinically significant genetic variations to guide treatment selection for both current and future patients [32]. One such test covering 324 genes, FoundationOne CDx (F1CDx), gained FDA approval and proposed coverage from The Centers for Medicare and Medicaid Services (CMS) in November 2017 [37].

The original draft of the national coverage determination (NCD) released with the F1CDx approval in November sought to outline all cases where an NGS test would be covered and propose non-coverage for any tests that failed to meet those requirements. The draft NCD proposed coverage for FDA-approved companion diagnostics and coverage with evidence development for FDA-approved non-companion diagnostics as well as non-FDA approved tests used as part of an NCI clinical trial. For tests covered with evidence development, the outcomes of sequenced patients had to be recorded in a prospective registry. The final NCD published in March 2018, however, eliminated the evidence development category and instead only provides national coverage for NGS tests with FDA approval or clearance as a companion diagnostic. All tests outside of this scope (tests that are either not FDA-approved or are not companion diagnostics) are left to the discretion of Medicare Administrative Contractors, which are private health care insurers with geographic jurisdiction to process Medicare claims.

## 4. Discussion

With the continued growth of NGS technology in oncology, two major questions loom large over the field. First, can the widely reported high percentage of actionable mutations in cancer cohorts translate into better patient outcomes? Second, should insurers cover the cost of genomic sequencing and sequencing-matched therapies, particularly in off-label settings?

To address the first question, we propose that studies reporting the percentage of actionable mutations should use transparent, precise, and commonly accepted definitions. Additionally, studies should stratify their results to give a clear indication of the existing evidence that the mutation will lead to an improvement in a patient’s care. Finally, more studies need to examine why so few patients with actionable mutations are receiving targeted therapy. For example, studies should evaluate the timing of sequencing in a patient’s care, the ability of physicians to interpret the results, and the accessibility and affordability of off-label treatment in clinical trials or otherwise.

These potential studies will also help answer the second question by uncovering the effect of insurance on patients’ utilization of and access to targeted therapies. Existing studies such as the Targeted Agent and Profiling Utilization Registry (TAPUR) Study and the NCI MATCH Study are both good examples of projects aiming to evaluate sequencing-driven cancer care [38,39]. These studies are increasingly important after CMS’s final NCD eliminated the coverage with evidence development category. By doing so, CMS relinquished a valuable opportunity to require at least some level of tracking of patient outcomes. With CMS stepping back from the creation of a prospective registry, it is increasingly important that TAPUR, NCI MATCH, and public-private partnerships, such as GA4GH [40], track patients’ overall survival, progression-free survival, response rate, and other data which will be useful for evaluating sequencing-matched therapies’ effect on patient outcomes and informing future treatment and insurance coverage standards.

Several stakeholders critical of CMS’s decision to cover F1CDx have emphasized the need for more evidence from trials like TAPUR and NCI MATCH to demonstrate the benefit of NGS tests beyond the current standard of testing specific actionable genes [41]. They argue that, aside from not having increased benefit, the additional genes sequenced in F1CDx may lead to increased spending and patient risk due to increased use of off-label sequencing-matched therapies [15,16,17,41]. Recent results from the NCI MATCH Study have shown modest benefits, reporting 0% [42], 8.1% [43], and 5% [44] objective response rates respectively for patients with mutations in the *PIK3CA* gene treated with taselisib, patients with overexpression of the protein HER2 treated with ado-trastuzumab emtansine, and patients with mutations in the FGFR pathways treated with the drug AZD4547. While these studies may uncover subpopulations where these drugs will be more effective, the overall numbers are underwhelming at present.

The willingness of insurers to cover sequencing-matched therapy is affected not only by outcomes, but also by cost. In the ongoing debate over health care, law makers should note the high cost of sequencing-matched therapies and establish policies for making these therapies and prescription drugs in general more affordable. One particular area of focus should be the oversight and regulation in pricing of drugs and efforts to increase price transparency, particularly for sequencing-matched therapies, which tend to be costly.

Without large, multi-institutional studies evaluating outcomes for patients getting off-label therapies, insurers are left to strike a balance between covering the latest treatments and preventing overly-optimistic and potentially harmful last-ditch treatment attempts. Leaving these policy decisions in the hands of insurers instead of medical associations and federal, state, and local regulatory agencies leads to highly variable care with some patients getting coverage for the exact same care that others do not.

We reported above on the promise of incorporating NGS into oncology care, while also highlighting the current shortcomings of putting this theory into practice. It is our hope that in doing so, research and attention will be directed appropriately to help maximize the benefit of precision medicine for cancer patients.

## Figures and Tables

**Table 1:** Summary of Outcomes in Oncology Precision Medicine Studies.

Study	Sample Size	Most Prevalent Tumor Types	Outcomes Reported
Tsimberidou et al. *Clin. Cancer Res.* **2012** [5]	291 patients with one molecular aberration (175 treated with matched therapy, 116 control)	Colorectal, melanoma, lung, ovarian	Matched group had improved ORR (27% vs. 5%), TTF (median 5.2 vs. 2.2 month), OS (median 13.4 vs. 9.0 month)
Radovich et al. *Oncotarget* **2016** [6]	101 patients with sequencing and follow up (44 treated with matched therapy, 57 control)	Soft tissue sarcoma, breast, colorectal	Matched group had improved PFS (86 vs. 49 days)
Schwaederle et al. *Mol. Cancer Ther.* **2016** [7]	180 patients with sequencing and follow up (87 treated with matched therapy, 93 control)	Gastrointestinal, breast, brain	Matched group had improved PFS (4.0 vs. 3.0 month), TRR (34.5% vs. 16.1% achieving SD/PR/CR)
Kris et al. *JAMA* **2014** [8]	578 patients with oncogenic driver and followup (260 with matched therapy, 318 control)	Lung only	Matched group had improved survival (median 3.5 vs. 2.4 years)
Aisner et al. *J. Clin. Oncol.* **2016** [9]	187 patients with targetable alteration and follow up (112 with matched therapy, 74 control)	Lung only	Matched group had improved survival (median 2.8 vs. 1.5 years)
Stockley et al. *Genome Med.* **2016** [10]	245 patients with sequencing matched to clinical trials (84 on matched trial, 161 control)	Gynecological, lung, breast	Matched group had improved ORR (19% vs. 9%)
LeTourneau et al. *Lancet Oncol.* **2015** [11]	RCT with 195 patients with molecular aberration (99 treated with matched therapy, 96 control)	Gastrointestinal, breast, brain	No difference in PFS between groups

ORR = overall response rate, TTF = time to treatment failure, OS = overall survival, PFS = progression free survival, TRR = tumor response rate, SD = stable disease, PR = partial response, CR = complete response, RCT = randomized controlled trial. Matched group indicates patients matched to a therapy based on sequencing results.

**Table 2:** The Percentage of Patients Receiving Matched Therapy. Summary of the number of patients with sequencing data, the number of patients with an actionable mutation, and the number of patients who go on to receive therapy matched to their sequencing results.

Study	Sample Size with Molecular Analysis	Sample Size with Actionable Mutation	Sample Size on Matched Therapy
Tsimberidou et al. *Clin. Cancer. Res.* **2012** [5]	1144	460 (40%)	211 (18%)
Radovich et al. *Oncotarget* **2016** [6]	101	NR	44 (44%)
Schwaederle et al. *Mol. Cancer. Ther.* **2016** [7]	347	NR	87 (25%)
Kris et al. *JAMA* **2014** [8]	999	617 (62%)	275 (28%)
Aisner et al. *J. Clin. Oncol.* **2016** [9]	919	529 (58%)	127 (14%)
Stockley et al. *Genome Med.* **2016** [10]	1640	938 (57%)	84 (5%)
LeTourneau et al. *Lancet Oncol.* **2015** [11]	496	293 (59%)	99 (20%)
Beltran et al. *JAMA Oncol.* **2015** [23]	97	91 (94%)	5 (5%)
Sohal et al. *J. Natl. Cancer. Inst.* **2015** [20]	233	109 (47%)	24 (10%)
Meric-Bernstam et al. *J. Clin. Oncol*. **2015** [21]	2000	789 (40%)	83 (4%)
Andre et al. *Lancet Oncol.* **2014** [22]	281	195 (69%)	55 (20%)

NR = not reported.
